# Striatal subdivisions that coherently interact with multiple cerebrocortical networks

**DOI:** 10.1002/hbm.24275

**Published:** 2018-07-05

**Authors:** Akitoshi Ogawa, Takahiro Osada, Masaki Tanaka, Masaaki Hori, Shigeki Aoki, Aki Nikolaidis, Michael P. Milham, Seiki Konishi

**Affiliations:** ^1^ Department of Neurophysiology Juntendo University School of Medicine Tokyo Japan; ^2^ Department of Radiology Juntendo University School of Medicine Tokyo Japan; ^3^ Research Institute for Diseases of Old Age, Juntendo University School of Medicine Tokyo Japan; ^4^ Sportology Center Juntendo University School of Medicine Tokyo Japan; ^5^ Center for the Developing Brain Child Mind Institute New York New York USA

**Keywords:** basal ganglia, caudate nucleus, putamen, resting‐state functional connectivity

## Abstract

The striatum constitutes the cortical‐basal ganglia loop and receives input from the cerebral cortex. Previous MRI studies have parcellated the human striatum using clustering analyses of structural/functional connectivity with the cerebral cortex. However, it is currently unclear how the striatal regions functionally interact with the cerebral cortex to organize cortical functions in the temporal domain. In the present human functional MRI study, the striatum was parcellated using boundary mapping analyses to reveal the fine architecture of the striatum by focusing on local gradient of functional connectivity. Boundary mapping analyses revealed approximately 100 subdivisions of the striatum. Many of the striatal subdivisions were functionally connected with specific combinations of cerebrocortical functional networks, such as somato‐motor (SM) and ventral attention (VA) networks. Time‐resolved functional connectivity analyses further revealed coherent interactions of multiple connectivities between each striatal subdivision and the cerebrocortical networks (i.e., a striatal subdivision‐SM connectivity and the same striatal subdivision‐VA connectivity). These results suggest that the striatum contains a large number of subdivisions that mediate functional coupling between specific combinations of cerebrocortical networks.

## 1 INTRODUCTION

The cerebral cortex and the striatum constitute a critical part of the cortical‐basal ganglia loop, in which information is processed through the cerebral cortex, basal ganglia, and thalamus and returns to the cerebral cortex (Alexander, DeLong, & Strick, 1986; Haber, 2003). The striatum has classically been discriminated into the caudate nucleus, which is connected with the prefrontal cortex, and the putamen, which is connected with the frontal motor cortex, and it is thought to select the most appropriate behavior by interacting with the cerebral cortex (Alexander et al., 1986; Haber, 2003; Middleton & Strick, 2000). Using resting‐state functional and structural connectivity (Behrens et al., 2003; Fox & Raichle, 2007; van den Heuvel & Pol, 2010), the precise architecture of the human striatum and its converging input from the cerebral cortex have been revealed by using clustering analyses based on the global similarity of functional/structural connectivity (Barnes et al., 2010; Choi, Yeo, & Buckner, 2012; Di Martino et al., 2008; Draganski et al., 2008; Garcia‐Garcia et al., 2018; Janssen, Jylänki, Kessels, & van Gerven, 2015; Jarbo & Verstynen, 2015; Jaspers, Balsters, Kassraian Fard, Mantini, & Wenderoth, 2017; Jung et al., 2014; Verstynen, Badre, Jarbo, & Schneider, 2012). Recent animal tracer studies have also revealed fine organization in the striatum, particularly in its rostral part, in relation to the converging input from the cerebral cortical areas (Averbeck, Lehman, Jacobson, & Haber, 2014; Choi, Ding, & Haber, 2017; Choi, Tanimura, Vage, Yates, & Haber, 2017; Haber, Kim, Mailly, & Calzavara, 2006). However, it remains unclear how the striatum functionally interacts with multiple cerebrocortical networks in the temporal domain. The central question of this study is to reveal the temporal aspects of the multiple striatal‐cerebrocortical interactions; for this purpose, it is important to analyze such interactions on the basis of the functionally distinct subdivisions.

The cerebral cortex has been divided into functional cerebrocortical areas (Bzdok et al., 2015; Eickhoff, Laird, Fox, Bzdok, & Hensel, 2016; Eickhoff, Thirion, Varoquaux, & Bzdok, 2015; Finn et al., 2015; Genon et al., 2017; Jackson, Bajada, Rice, Cloutman, & Lambon Ralph, 2018; Jakobsen et al., 2018; Mars et al., 2011; Shen, Tokoglu, Papademetris, & Constable, 2013; Wang, Fan, et al., 2015; Wang, Yang, et al., 2015; Wang et al., 2017; Zhang & Li, 2012; Zhang et al., 2016). There are two major approaches for areal parcellation (Schaefer et al., 2017): Clustering analyses reveal cortical areas that represent the global cortical functional architecture well. In contrast, boundary mapping analyses detect boundaries based on local gradients of connectivity profiles. It has been suggested that the local gradient approach is more suitable for delineating cortical areas because detecting abrupt changes in connectivity profiles is similar to the histological delineation of cortical areas (Barnes et al., 2010; Biswal et al., 2010; Cohen et al., 2008; Glasser et al., 2016; Gordon et al., 2016; Gordon, Laumann, Gilmore, et al., 2017; Hirose et al., 2012, 2013, 2016; Laumann et al., 2015; Margulies et al., 2007; Nelson et al., 2010; Poldrack et al., 2015; Schaefer et al., 2017; Wig et al., 2014; Wig, Laumann, & Petersen, 2014; Xu et al., 2016). Therefore, it is expected that boundary mapping analyses are more suitable for the stable detection of smaller striatal subdivisions and that boundary mapping analyses can reveal the temporal aspects of the striatal‐cortical interactions more accurately.

In the present functional MRI study, boundary mapping analyses were applied to the striatum to obtain the finer functional architecture of the human striatum. We delineated approximately 100 striatal subdivisions reproducibly across independent data sets. Based on previous studies of converging input from the cortex, it was expected that each striatal subdivision was connected with multiple cerebrocortical networks. We next examined how the striatum interacted with the cerebrocortical networks by analyzing connectivity changes over time (Hutchison et al., 2013; Shine et al., 2015, 2016; Zalesky, Fornito, Cocchi, Gollo, & Breakspear, 2014) to reveal whether the multiple interactions between a striatal subdivision and multiple cortical networks are coherent or independent.

## 2 MATERIALS AND METHODS

### 2.1 Participants

Ten right‐handed healthy young participants [six males and four females, 27.0 ± 7.7 years old (mean ± *SD*), range 20–39] participated in the experiments. Written informed consent was obtained from all of the participants according to the Declaration of Helsinki. The experimental procedures were approved by the Institutional Review Board of Juntendo University School of Medicine.

### 2.2 MRI procedures

All MRI data were acquired using a 3‐T MRI scanner (Siemens Skyra, Erlangen, Germany). T1‐weighted structural images were obtained for anatomical reference (resolution = 0.8 × 0.8 × 0.8 mm^3^). Functional images were obtained using multiband gradient‐echo echo‐planar sequences (Feinberg et al., 2010; Moeller et al., 2010; Xu et al., 2013; TR = 4.0 s, TE = 41.6 ms, flip angle = 73º, FOV = 160 × 160 mm^2^, matrix size = 128 × 128, 120 contiguous slices, voxel size = 1.25 × 1.25 × 1.25 mm^3^, multiband factor = 4). We acquired 100 volumes in each fMRI run at the resting state and repeated the process for 10 runs in each of the 10 daily sessions. Thus, 10,000 total volumes were collected for each participant. Eight of the 10 participants also participated in a task fMRI experiment and were scanned for six runs using the same scanning parameters.

To increase the multiband factor, a small FOV (160 × 160 mm^2^) was set. This small FOV did not always cover the whole brain along the anterior–posterior axis. However, the aliasing artifacts in the frontal lobe (from the occipital lobe) were minimal, and the aliasing overlap artifact observed in the frontal lobe was 0.1%–4.9% of the whole cerebral cortex, keeping the striatum intact. It has also been demonstrated that parcellation procedures do not require the entire cerebral cortex for the appropriate detection of the connectivity transitions (Hirose et al., 2012, 2013). Therefore, it is unlikely that the FOV influenced either the signal acquisition in the striatum or the data analyses used in the present study.

### 2.3 Parcellation of the striatum

Functional images were preprocessed for resting‐state functional connectivity (Fox et al., 2005; Fox & Raichle, 2007). Images were corrected for slice timing and realigned using SPM8 (r4290) (http://www.fil.ion.ucl.ac.uk/spm). Temporal filters (0.009 Hz < *f* < 0.08 Hz) were applied to the functional images using FSL (ver. 5.0.6) (Smith et al., 2004). A general linear model (Worsley & Friston, 1995) was used to regress out nuisance signals that correlated with head motion, whole‐brain global signals, averaged ventricular signals and averaged white matter signals.

**Figure 1:**
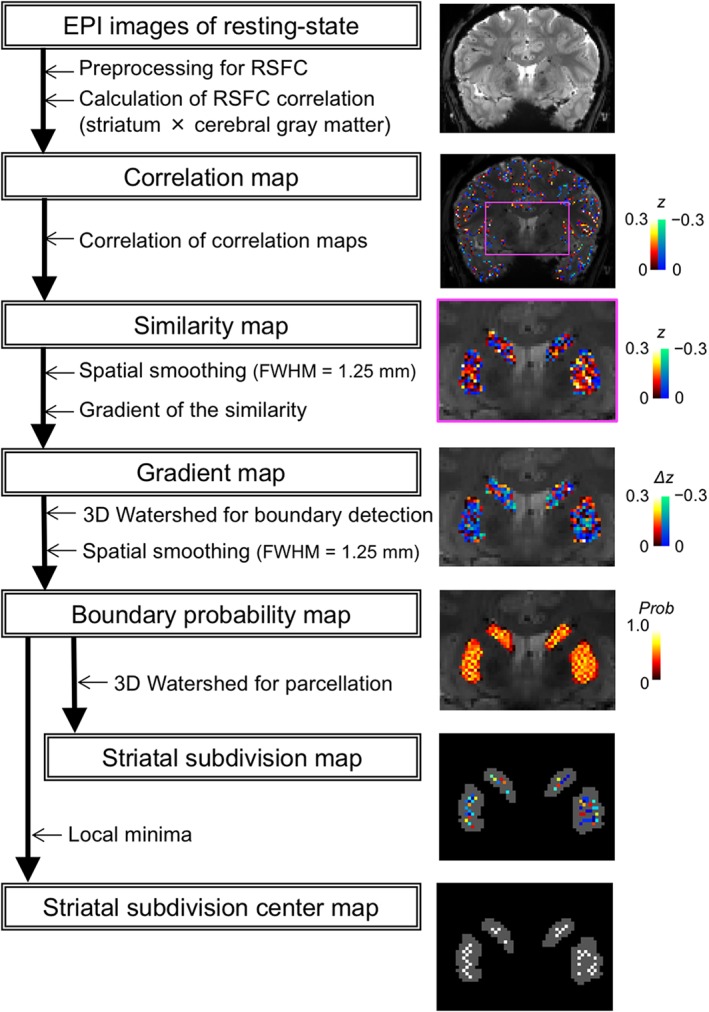
Overview of boundary mapping analyses of the striatum. functional images were processed, and the correlation, similarity, gradient, boundary probability, and striatal parcel center maps were generated as shown for the analysis flow. Representative examples for these maps are shown in the right plots

Parcellation analyses based on boundary mapping (Cohen et al., 2008; Glasser et al., 2016; Gordon et al., 2016; Gordon, Laumann, Gilmore, et al., 2017; Hirose et al., 2012, 2013, 2016; Laumann et al., 2015; Osada et al., 2017) were applied to the striatum (Figure 1). Each voxel in the striatum of each participant was used as a seed to calculate its correlations with the voxels in the gray matter of the cerebral cortex. One part of the cerebral cortex that suffered from overlap due to aliasing artifacts was removed from the target voxels. Voxel‐wise correlation coefficients in the cerebral cortex were converted to Fisher's *z* (Fisher *z* transformation).

The similarity of the spatial patterns of the correlation maps was then evaluated using correlation coefficients, and similarity maps were generated. After minimal spatial smoothing (full width at half‐maximum [FWHM] = 1.25 mm), spatial gradients of the similarity maps were computed for each seed voxel. A three‐dimensional watershed algorithm (Vincent & Soille, 1991) was applied to the gradient maps, and the binary watershed maps were averaged across seed voxels after spatial smoothing (FWHM = 1.25 mm) to generate a boundary probability map. The watershed algorithm was again applied to the boundary probability map for each participant. A cluster with three or more contiguous voxels was defined as a striatal subdivision, and the voxel with the smallest boundary probability in the striatal subdivision was defined as a striatal subdivision center (SSC).

The striatum was manually segmented using the functional image of each participant. The border of the striatum was clear for the most part, but the ventral surface of the ventral striatum that was relatively obscure was determined using the T1‐weighted structural images as reference. It should be noted that the boundary mapping of the ventral striatum is relatively less sensitive for detecting striatal subdivisions. The striatum analyzed in the present study includes both the dorsal and ventral striatum. However, the ventral striatum is much smaller in size than is the dorsal striatum, and the outermost voxels of the ventral striatum, which are not detected as centers of the subdivisions using boundary mapping analyses, occupy a large portion of the ventral striatum.

### 2.4 Parcellation of the cerebral cortex

Functional images were preprocessed for resting‐state functional connectivity in the same way as the striatum (see section 2.3). For volume to surface mapping, the middle of the gray matter was identified within a functional image for each participant using Caret software (Marcus et al., 2011; Van Essen et al., 2001), and a fiducial surface image was generated from the middle of the gray matter.

The cerebrocortical parcels were calculated in a similar manner, as described previously (Cohen et al., 2008; Gordon et al., 2016; Laumann et al., 2015; Supporting Information Figure S1). Each vertex in the fiducial surface in the cerebral cortex of each participant was used as a seed to calculate its correlations with all of the vertices. The similarity of the spatial patterns of the correlation maps was then evaluated using correlation coefficients, and similarity maps were generated. After spatial smoothing (FWHM = 6.0 mm) (Gordon et al., 2016; Laumann et al., 2015), spatial gradients of the similarity maps were computed for each seed vertex. A two‐dimensional watershed algorithm was applied to the gradient maps, and the binary watershed maps were averaged across the seed vertices after spatial smoothing (FWHM = 6.0 mm) to generate a boundary probability map. The watershed algorithm was again applied to the boundary probability map to delineate cerebrocortical parcels for each participant. The voxel with the smallest boundary probability in the cerebrocortical parcel was defined as a cerebrocortical parcel center.

### 2.5 Quality control of head movement

We evaluated the amount of head motion by using frame‐wise displacement (FD) (Power, Barnes, Snyder, Schlaggar, & Petersen, 2012). FD is a measurement of instantaneous head motion that can be calculated as a locational difference between two successive volumes and is an important measure for the quality control of resting‐state data (Gordon et al., 2016; Laumann et al., 2015). Frames with FD > 0.2 mm were censored, as well as uncensored segments of data lasting fewer than five contiguous volumes; all such data were excluded from the subsequent analysis. The included images were 75.1 ± 12.6% (mean ± *SD*) of the total acquired images, and the resultant FD was 0.084 ± 0.012 mm (mean ± *SD*).

### 2.6 Evaluation of the relationship between the striatal subdivisions and cerebrocortical parcels

To evaluate whether the striatal subdivisions and cerebrocortical parcels were related to each other, the voxel‐wise variation of connectivity with the cerebrocortical parcels was examined within each striatal subdivision. For each center voxel of the cerebrocortical parcels, Fisher's *z* value was calculated with all of the voxels in the striatum; then the *SD* of the *z* values was calculated within each striatal subdivision. As a control, the pattern of the striatal subdivisions was shifted by one voxel along 26 directions from the original position, and the *SD*s of Fisher's *z* values in the shifted striatal subdivisions were calculated in a similar way. The indeterminable voxels between the striatum and its surrounding white matter/cerebrospinal fluid were not included in the striatal subdivisions or in their boundaries. The *SD* indicates how uniformly the voxels in a striatal subdivision are connected with each cerebrocortical parcel center.

### 2.7 Replication of larger‐scale functional organization using public atlases

We replicated previous observations on the larger‐scale architecture of the striatal connectivity with cortical gyri. Individual cortical surfaces were converted to the cerebrocortical surface atlas (Yeo et al., 2011) by Freesurfer (Fischl, Sereno, Tootell, & Dale, 1999), and multimodal surface matching (MSM; Robinson et al., 2014) was then applied. The correlations of each SSC with all of the voxels in the precentral gyrus (PCG), middle frontal gyrus (MFG), orbitofrontal gyrus (OFG), and inferior parietal lobule (IPL) (Desikan et al., 2006) were calculated, and the Fisher's *z* values across all of the voxels within each gyrus were averaged. The gyrus that was connected most strongly with each of the SSCs was determined out of the four gyri, and four SSC binary maps were generated that showed the SSCs that were most strongly connected with each gyrus. The binary SSC maps were averaged across participants for each gyrus, with spatial smoothing by a 4‐mm FWHM kernel. The 4‐mm kernel was chosen to investigate the striatum on a larger scale, but the kernel was kept smaller than the 6‐mm kernel applied to the cerebral cortex. The resultant values for the smoothed images were normalized such that the total values across the entire striatum were 1.

Similarly, correlations of each SSC with the seven networks including the somato‐motor (SM), ventral attention (VA), limbic (Lim), and fronto‐parietal (FP) (Yeo et al., 2011) were calculated. The network that was connected most strongly with each of the SSCs was determined out of the seven networks, and seven SSC binary maps were generated to show the SSCs that were most strongly connected with each network. The binary SSC maps were averaged across participants for each network, with spatial smoothing by a 4‐mm FWHM kernel.

### 2.8 Dynamic functional connectivity analysis

We determined the two of the seven cerebrocortical networks that were connected most strongly with each SSC. Most of the striatal subdivisions were connected with the specific combinations of cerebrocortical networks, primarily SM‐VA and VA‐FP combinations. To investigate whether the multiple interactions were temporally coherent, we used a dynamic functional connectivity analysis (Hutchison et al., 2013) using multiplication of temporal derivatives (MTD), a sensitive index of functional connectivity within a small time‐window (Shine et al., 2015, 2016). The MTD allows greater temporal resolution of time‐resolved connectivity in BOLD time series data than does the conventional sliding‐window Pearson's correlation coefficient (Shine et al., 2015).

Functional images were preprocessed for dynamic functional connectivity analysis (Leonardi & Van De Ville, 2015; Shine et al., 2015, 2016). Images were corrected for slice timing and realigned using SPM8 (http://www.fil.ion.ucl.ac.uk/spm). Temporal filters (0.05 Hz < *f* < 0.125 Hz) were applied to the functional images. A general linear model was used to regress out nuisance signals that correlated with head motion, whole‐brain global signals, averaged ventricular signals, and averaged white matter signals. Time series data were extracted from each voxel in the SM, VA, and FP networks (Yeo et al., 2011) and from each SSC that was connected most strongly with the SM‐VA or VA‐FP network combination. The MTD between the striatal subdivisions and the cerebrocortical networks was calculated based on the sliding window of 7 TRs, with a step size of 1 TR. The MTD was then averaged across the voxels in each cerebrocortical network and further across the voxels of the SSCs that were connected most strongly with the SM‐VA or VA‐FP network.

## 3 RESULTS

### 3.1 Striatal subdivisions

**Figure 2:**
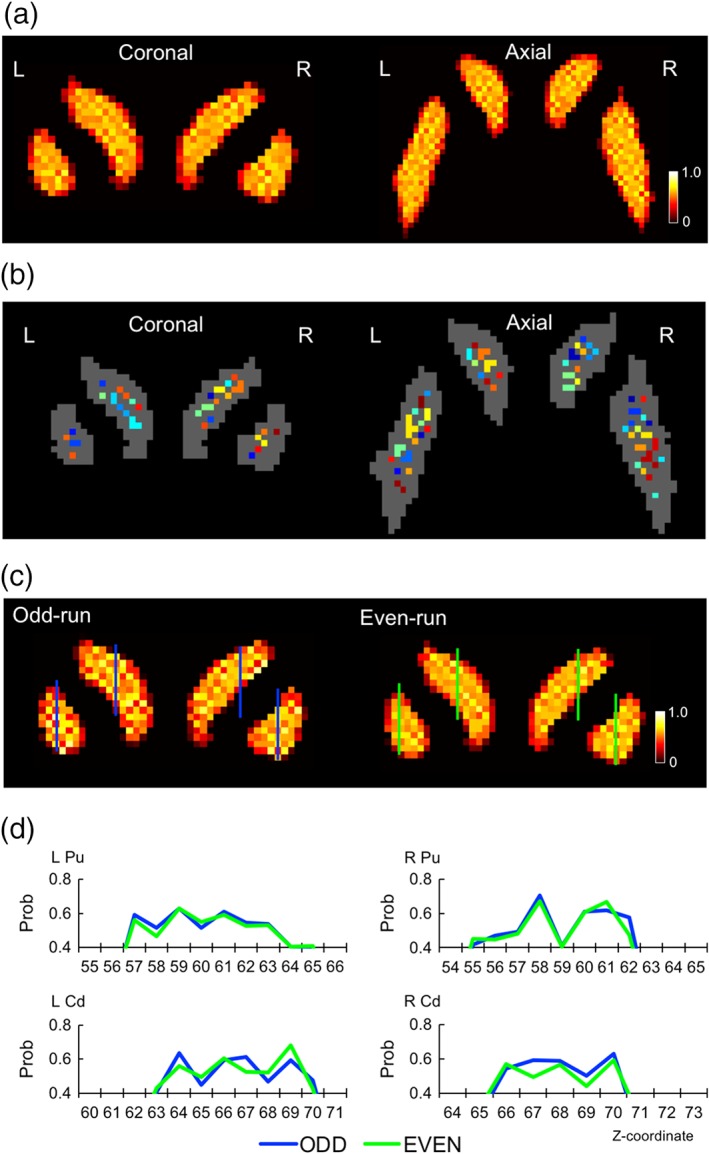
Striatal parcellation results. (a) Boundary probability maps shown in coronal and axial slices calculated from the entire data set of one participant. The color scale indicates the probability of boundaries as determined by a watershed algorithm. (b) Striatal subdivisions are coded in different colors. (c) The reproducibility of the probability maps was evaluated by dividing the data sets into odd and even runs. (d) Spatial profiles of the probabilistic boundary patterns generated from two independent data sets (odd/even runs). Pu: Putamen, Cd: Caudate nucleus

The parcellation analysis (Figure 1) revealed the boundary probability map (Figure 2a; see also Supporting Information Figure S2a for all participants) in the original space of each participant that contained approximately 100 (94.7 ± 24.0, mean ± *SD*) striatal subdivisions in the brain (Figure 2b). In a group analysis with fine normalization using DARTEL (Ashburner, 2007), the boundary pattern in the striatum was almost lost (Supporting Information Figure S2b). The boundary probability map was highly reproducible between independent data sets, when the total data sets were divided into odd or even runs (Figure 2c) with a correlation coefficient for each participant of .90 (Fisher's *z* = 1.53) [t(9) = 16.4, *p* < .001 after *z* transformation] (see Table 1 for all participants). When the high spatial frequency components (≤2.5 mm) of the probability map that constituted the boundary pattern were extracted using Fourier transformation, the average correlation coefficient of the high‐frequency component was .37 (Fisher's *z* = 0.39) [t(9) = 7.2, *p* < .001 after *z* transformation]. Figure 2d demonstrates spatial profiles of the probabilistic boundary patterns that exhibited a close match across the two independent data sets. As shown in the distribution of the distance between adjacent striatal subdivisions in Supporting Information Figure S3, the size of the striatal subdivisions was reproducible across participants. The distribution of the size was also similar between the left and right striatum. In almost all of the participants, the most common distance between adjacent subdivisions was 2 mm.

**Table 1:** Fisher's *z* values and correlation coefficients (in parenthesis) of correlation between two striatal probability maps (P‐maps) and between two cortical correlation maps (Z‐maps)

Participant	P‐maps	Z‐maps	Z‐maps
	Odd/even	Odd/even	Nearest SSCs
1	1.16 (0.82)	0.34 (0.33)	0.021 (0.021)
2	1.83 (0.95)	0.56 (0.51)	0.058 (0.058)
3	1.65 (0.93)	0.50 (0.46)	0.002 (0.002)
4	1.38 (0.88)	0.58 (0.52)	0.018 (0.018)
5	1.74 (0.94)	0.48 (0.45)	−0.003 (−0.003)
6	1.83 (0.95)	0.51 (0.47)	0.045 (0.045)
7	1.29 (0.86)	0.50 (0.46)	−0.005 (−0.005)
8	1.94 (0.96)	0.51 (0.47)	0.027 (0.027)
9	1.33 (0.87)	0.60 (0.54)	0.009 (0.009)
10	1.16 (0.82)	0.45 (0.42)	0.000 (0.000)
Average	1.53 (0.90)	0.50 (0.46)	0.017 (0.017)
*t* value	16.4	21.0	2.55
*p* value	< .001	< .001	< .05

### 3.2 Relationship of the striatal subdivisions with cerebrocortical parcels

**Figure 3:**
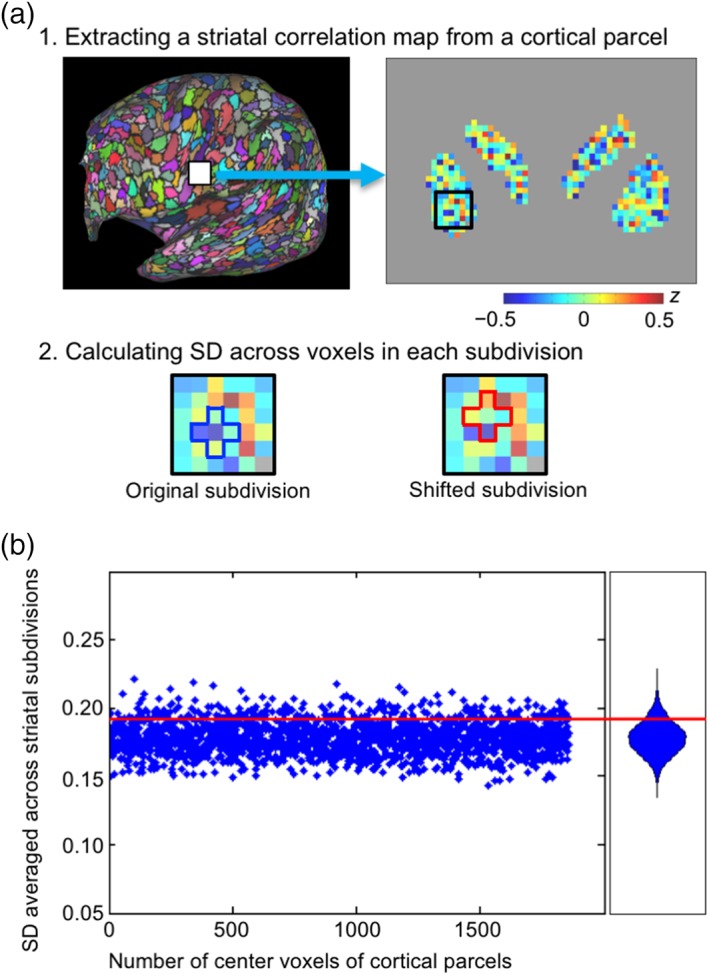
Connectivity between striatal subdivisions and cerebrocortical parcels. (a) Image processing stream for the data presented in Figure 3b. A striatal correlation map of Fisher's *z* values was generated in one participant when the seed was taken from a cerebrocortical parcel center. The across‐voxel *SD* of Fisher's *z* values in each striatal subdivision were then calculated. (b) Distribution of the across‐voxel *SD*s of correlations between striatal voxels and cerebrocortical parcel centers in the striatal subdivisions of one participant. A blue dot indicates the across‐voxel *SD* of correlations from one cerebrocortical parcel, averaged across all of the striatal subdivisions. A red line indicates the across‐voxel *SD* of correlations averaged across the shifted striatal subdivisions, again averaged across the cerebrocortical parcels

We examined how the striatum was related to the cerebrocortical parcels at the subdivision level by calculating the across‐voxel *SD* of Fisher's *z* value of the striatal correlation map within each striatal subdivision (Figure 3a). Figure 3b shows the distribution of the *SD*s of cortex‐striatum correlation coefficients within the subdivisions (blue dots) and the average *SD* within the shifted the subdivisions as a control (red line) in one participant. The distribution of the *SD*s was significantly lower than the control [group average *SD* = 0.175, group average *SD* (control) = 0.188, t(9) = 19.1, *p* < .001], which suggests that the cerebrocortical parcels are more likely connected uniformly within the striatal subdivisions.

### 3.3 Intricate organization of the striatal subdivisions connected with the cerebral cortex

**Figure 4:**
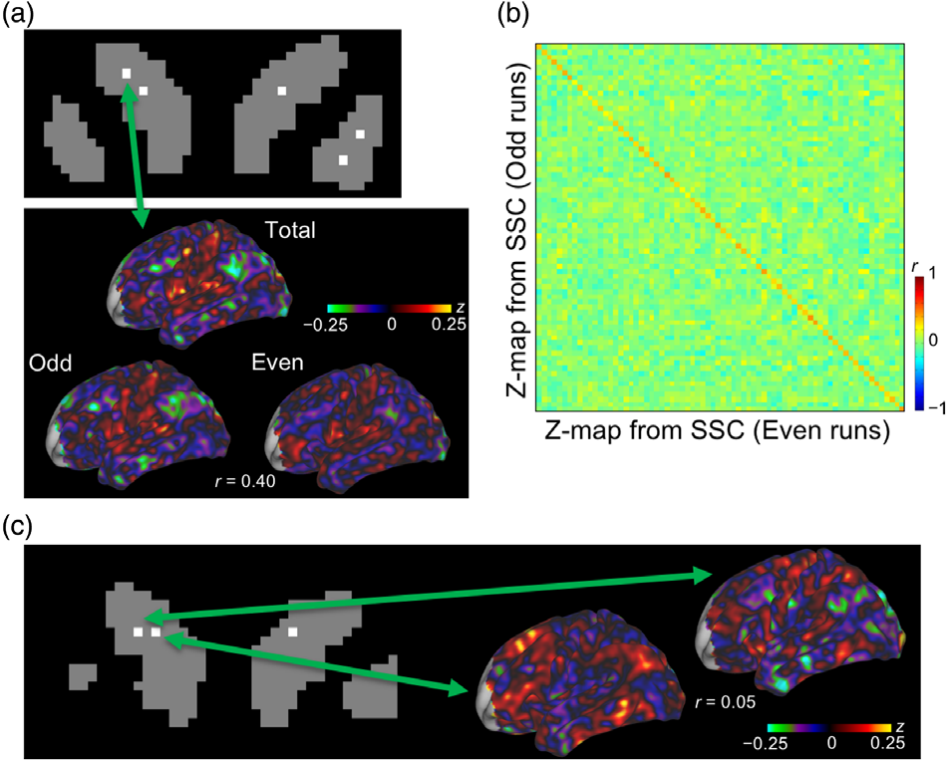
Connectivity of the striatal subdivisions with the cerebral cortex. (a) Cerebrocortical correlation maps of one participant calculated from total, odd or even runs, when the seed was taken from one voxel of the striatal subdivision center (SSC). The color scale indicates Fisher's *z* values of the correlation. (b) A correlation matrix of the cerebrocortical correlation maps when the seed was taken from different SSCs. Correlations were calculated between the cerebrocortical correlation maps from each of the SSCs based on odd and even runs. The color scale indicates the correlation coefficients between Fisher's *z* maps. (c) Cerebrocortical correlation maps when the seeds were taken from the nearest pair of SSCs

We next examined the functional connectivity of striatal subdivisions with the cerebral cortex. The SSC was defined as the voxel with the smallest boundary probability value in a subdivision, and the functional connectivity was calculated with the SSC voxels as seeds. The functional connectivity with the cerebral cortex was significantly reproducible between the two independent data sets, when the overall data sets were divided into odd or even runs (Figure 4a), with an average correlation coefficient of .46 (Fisher's *z* = 0.50) [t(9) = 21.0, *p* < .001 after *z* transformation] (Table 1). Figure 4b shows a correlation matrix of SSCs in the odd/even runs in the same participant, demonstrating high reproducibility between cerebrocortical correlation maps from the same subdivisions along the diagonal line.

The similarity of cerebrocortical correlation maps was then examined from adjacent (2.36 voxels or 2.95 mm apart, on average) striatal subdivisions (Figure 4c). Although the similarity of correlation maps was barely significant only in group statistics [t(9) = 2.55, *p* = .03 after *z* transformation], the average correlation coefficient was very small (*r* = 0.017, Fisher's *z* = 0.017), with negative correlation values in two of the participants (Table 1).

### 3.4 Larger‐scale functional organization of the striatum

Previous studies of striatal functional architecture have revealed a well‐organized relationship for cerebrocortical‐striatal connectivity (Barnes et al., 2010; Choi et al., 2012; Di Martino et al., 2008; Draganski et al., 2008; Garcia‐Garcia et al., 2018; Janssen et al., 2015; Jaspers et al., 2017; Jung et al., 2014). We confirmed, as a positive control, that the larger‐scale observations could be replicated using the present data set. Supporting Information Figure S4a shows group average maps of the SSCs that were most strongly connected with the PCG, MFG, OFG, or inferior parietal lobule, based on the atlas by Desikan et al. (2006). Consistent with previous studies (Barnes et al., 2010; Choi et al., 2012; Di Martino et al., 2008; Draganski et al., 2008; Garcia‐Garcia et al., 2018; Janssen et al., 2015; Jaspers et al., 2017; Jung et al., 2014), the middle part of the putamen was most strongly connected with the PCG, the caudate nucleus was most strongly connected with the MFG, and the caudate nucleus extending to ventral striatum was most strongly connected with the OFG.

Similarly, Supporting Information Figure S4b shows group average maps of SSCs that were connected most strongly with one of the seven cerebrocortical networks, including the SM, VA, Lim, and FP networks, as reported previously (Yeo et al., 2011). Consistent with a previous study (Choi et al., 2012), the ventral part of the putamen was most strongly connected with the SM network, the dorsal part of the putamen was connected most strongly with the VA network, the caudate nucleus was most strongly connected with the FP network, and the caudate nucleus extending to the ventral striatum was most strongly connected with the Lim network. These results confirmed the larger‐scale functional organization of the striatum.

### 3.5 Striatal subdivisions connected with multiple cerebrocortical networks

**Figure 5:**
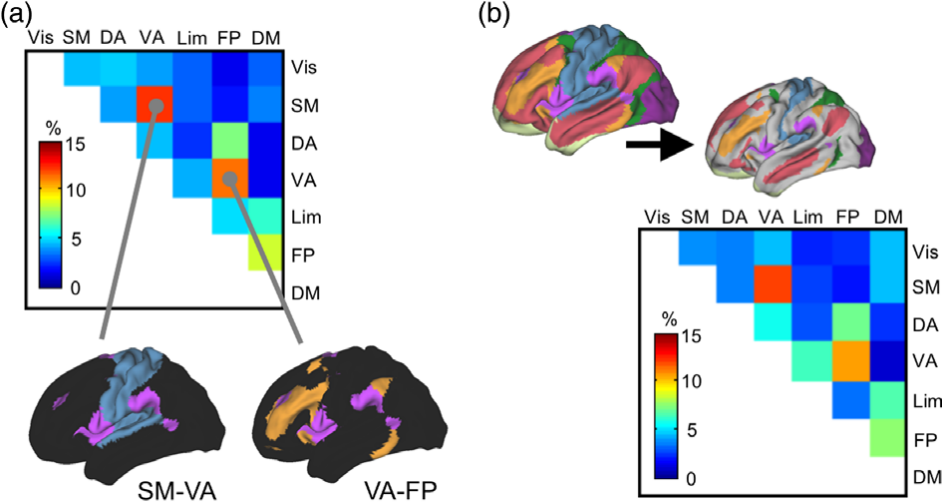
Combinations of cerebrocortical networks connected with the striatal subdivisions. (a) The distribution of combinations of the two cerebrocortical networks (of seven total networks) that were connected the most strongly and second most strongly with each striatal subdivision. The subdivisions were excluded from analysis when the second strongest connectivity was less than 30% of the strongest connectivity. The color scale indicates the percentage of each combination averaged across participants. SM: somato‐motor; VA: ventral attention; FP: fronto‐parietal; DM: default mode. (b) The distribution of network combinations when the size of the networks was reduced to approximately one half of the original size

We then examined how the striatal subdivisions were convergently connected with the two cerebrocortical networks. When the second strongest connectivity was less than 30% of the strongest one, the SSC was excluded from the analysis, resulting in exclusion of 21.2% of the total SSCs. Figure 5a shows the combinations of the two cerebrocortical networks with which the SSCs were connected most strongly. The most common combination (12.2%) was the SM‐VA networks, and the distribution of the combinations was highly biased toward specific combinations, such as the SM‐VA and VA‐FP networks [χ^2^ test, χ^2^(20) = 35.2, *p* < .019].

It has been demonstrated that the spatial extent of the DM network is variable among individuals (Braga & Buckner, 2017). To test whether the individual variability affected the distribution of the combinations of the connected networks, the regions that were likely to vary across individuals were excluded from analysis by removing the outermost vertices of the seven networks repeatedly until the resultant size of the networks was reduced to approximately one half of the original size (Figure 5b). The distribution of the combinations was almost unchanged (Figure 5b), which indicates that the biased distribution was not due to individual differences in the spatial extent of the networks.

To further examine the distribution of the combinations in a more conservative way, 37.4% and 62.2% of the total SSCs were excluded from analysis when the second strongest connectivity was less than 50% and 70% of the strongest one. The distribution of the combinations was almost unchanged (Supporting Information Figure S5a). The distribution was again almost unchanged when the size of the networks was reduced to approximately one half of the original size (Supporting Information Figure S5b).

### 3.6 Striatal cerebrocortical interaction revealed by dynamic functional connectivity

**Figure 6:**
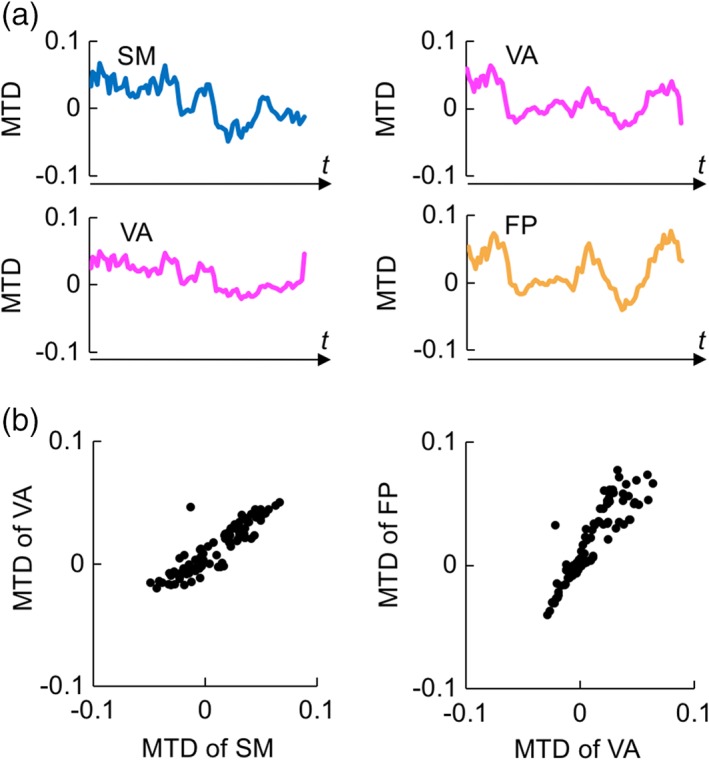
Dynamic functional connectivity between striatal subdivisions and cerebrocortical networks. (a) Time courses of MTD during the resting state calculated between the striatal subdivisions and SM/VA networks (left) and between the striatal subdivisions and VA/FP networks (right) in one run of one participant. The time courses were averaged across the subdivisions connected with the same set of cerebrocortical networks. MTD is shown in an arbitrary unit. (b) Scatter plots of the MTDs shown in plot 6a. One dot indicates MTD from one sliding window. MTD, multiplication of temporal derivatives

Striatal subdivisions were likely connected with multiple cerebrocortical networks such as the SM‐VA and VA‐FP networks. Figure 6a (left) shows the time courses of MTD (Shine et al., 2015, 2016) between the SM/VA network and the related SSCs in one representative run of one participant. The time courses were averaged across voxels in each cerebrocortical network and further averaged across the related SSCs. Figure 6a (right) shows the time courses of MTD between the VA/FP networks and the related SSCs in the same run. To examine the relationship between the two SSC‐cerebrocortical network interactions, the MTDs from each sliding window were plotted (Figure 6b). There was significant positive correlation between SSC‐SM and SSC‐VA [mean = 0.77 (Fisher's *z*), t(9) = 11.6, *p* < .001] and between SSC‐VA and SSC‐FP [mean Fisher's *z* = 0.40, t(9) = 7.84, *p* < .001], which suggested that temporal changes in the connectivity between SSC‐SM and SSC‐VA and between SSC‐VA and SSC‐FP were temporally coherent.

### 3.7 Brain activation during eye movements

We also measured the brain activity when participants performed a standard eye movement task (Supporting Information Text S1 and Figure S6a). Although a group analysis revealed activations of both the frontal eye field and the striatum, a single‐level analysis revealed activations only in the frontal eye field (Supporting Information Figure S6b). The lack of activation in the striatum at this single‐level analysis is consistent with a study by the Human Connectome Project (Barch et al., 2013; Van Essen et al., 2013), which reported a similar phenomenon.

## 4 DISCUSSION

In the present study, approximately 100 subdivisions were revealed using boundary mapping analyses applied to functional images of the striatum. The cerebrocortical parcels were more likely connected uniformly within the striatal subdivisions. The spatial organization of the striatum was intricate, in that adjacent striatal subdivisions showed only faintly similar correlation patterns with the cerebral cortex, although a larger scale organization was consistently observed. The members of the cerebral cortical networks connected with the striatal subdivisions were biased toward specific combinations such as SM‐VA and VA‐FP networks. Dynamic connectivity analyses revealed that the multiple interactions temporally changed coherently. These results suggest that the striatum contains a large number of subdivisions that integrate the functions of specific cerebrocortical networks in a temporally coherent manner.

It has been demonstrated that the cerebrocortical parcels show homogeneity within their regions (Gordon et al., 2016). The homogeneity is estimated by the greater proportion of the principal component of functional connectivity profiles against a null hypothesis using shifted boundaries. The homogeneity is demonstrated when the number of surface vertices within a parcel is sufficiently great (see figure 3 in Gordon et al., 2016). Thus, the homogeneity analysis was not applicable to the small structure of the striatum, and it is not certain whether the parcellated striatal areas can be considered as “striatal parcels.” On the other hand, the striatal subdivisions were highly reproducible when the data sets were divided into two halves. Moreover, the boundary pattern of the striatal subdivisions produced smaller across‐voxel *SD* of functional connectivity with the cerebrocortical parcels. Therefore, although a much higher spatial resolution would be required to demonstrate the “striatal parcels,” it is possible that the boundaries of the striatal subdivisions presented in this study reflects functional differences in the areas separated by the boundary. It has been shown that striatum contains chemical compartments called “striosomes” that are approximately 0.5–0.8 mm in size in humans (Graybiel & Ragsdale, 1978). Although it seems unlikely that the striatal subdivisions reported in the present study simply correspond to the striosomes, future investigations might reveal that multiple striosomes or a complex of a striosome and the surrounding compartment, called the “matrix,” are functionally related to striatal subdivisions.

The cerebral cortex can be parcellated into many functional areas in individual brains based on functional connectivity (Airan et al., 2016; Glasser et al., 2016; Gordon, Laumann, Adeyemo, Gilmore, et al., 2017; Gordon, Laumann, Adeyemo, & Petersen, 2017; Gordon, Laumann, Gilmore, et al., 2017; Laumann et al., 2015; Poldrack et al., 2015; Wang, Buckner, et al., 2015; Wang, Fan, et al., 2015; Wang, Yang, et al., 2015). To investigate individual differences, however, the individual brains have to be aligned to a common atlas. Although the alignment of the cerebral cortex was successful, even fine normalization using DARTEL was not successful in the striatal parcellation results (Supporting Information Figure S2). Thus, normalization using multimodal features such as structure and functional connectivity, as implemented in MSMAll (Glasser et al., 2016; Robinson et al., 2014) for the cerebral cortex, seems to be required for the striatum.

The analyses of the present study can be applied to various striatal‐cortical interactions. For example, Zhang, Ide, and Li (2012) examined the functional connectivity of three motor areas (SMA, aPreSMA, and pPreSMA) with the subcortical structures including the striatum and revealed a differential pattern of functional connectivity within the striatum when the seed was moved along the y axis. The analyses of the present study may reveal more refined architecture of the striatal‐cortical connections by examining the patterns of functional connectivity between the subdivisions and the cortical parcels in these motor areas. Such analyses may also be used to investigate whether the multiple striatal‐cortical interactions from these motor areas are temporally coherent or independent.

Although the similarity of correlation maps from adjacent striatal subdivisions (Figure 4) was barely significant in the group statistics, the average correlation coefficient was very small (Fisher's *z* = 0.017), suggesting that the functional organization of the striatum is rather intricate when viewed at the scale of the striatal subdivisions. At the same time, the larger scale organization of the striatum from the same data (Supporting Information Figure S4) was consistent with that found in previous studies due to larger spatial smoothing and group averaging. Animal tracer studies have demonstrated the convergence of projections from multiple cortical areas to the striatum (Averbeck et al., 2014; Choi, Ding, et al., 2017; Choi, Tanimura, et al., 2017; Haber et al., 2006). Human studies using structural and functional connectivity have also provided consistent evidence of converging projections (Barnes et al., 2010; Choi et al., 2012; Di Martino et al., 2008; Draganski et al., 2008; Janssen et al., 2015; Jarbo & Verstynen, 2015; Jaspers et al., 2017; Jung et al., 2014; Verstynen et al., 2012). The present study further suggests that many of the striatal subdivisions are connected with multiple cerebrocortical networks that consist of multiple cortical areas. The combinations of the cerebrocortical networks connected with the striatal subdivisions were not equally distributed but were restricted to specific networks, excluding the possibility that striatal subdivisions equally cover all of the combinations of cerebrocortical networks, as “hypercolumns” do (Hubel, 1982). For example, it is possible that the sensory and motor processing of the SM network might trigger the bottom‐up attentional processing of the VA network, through input from one of the networks and output to both of the networks, via the cortical basal ganglia loop. Although the precise interpretation of functional connectivity results may require further investigation, the present study revealed approximately 100 striatal subdivisions that may mediate the functional integration of cerebrocortical networks.

## CONFLICT OF INTEREST

The authors have no conflict of interest to declare.

## Supporting information


**Appendix S1**: Supporting Information
