# Supplementary material for: Striatal subdivisions that coherently interact with multiple cerebrocortical networks
Source: Hum Brain Mapp. 2018 Jul 5;39(11):4349–59. doi: 10.1002/hbm.24275 (PMC6220841; doi:10.1002/hbm.24275)
Supplement: Supplementary file 1 — Appendix S1: Supporting Information [file HBM-39-4349-s001.docx]

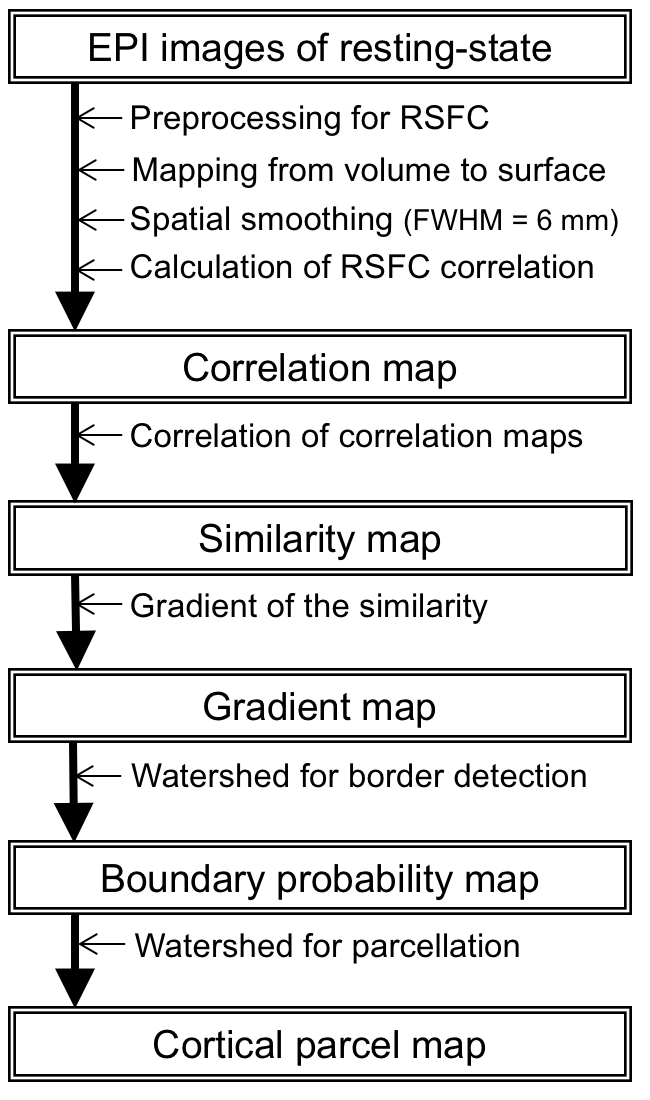


**Figure S1**  Overview of boundary mapping analyses of the cerebral cortex.


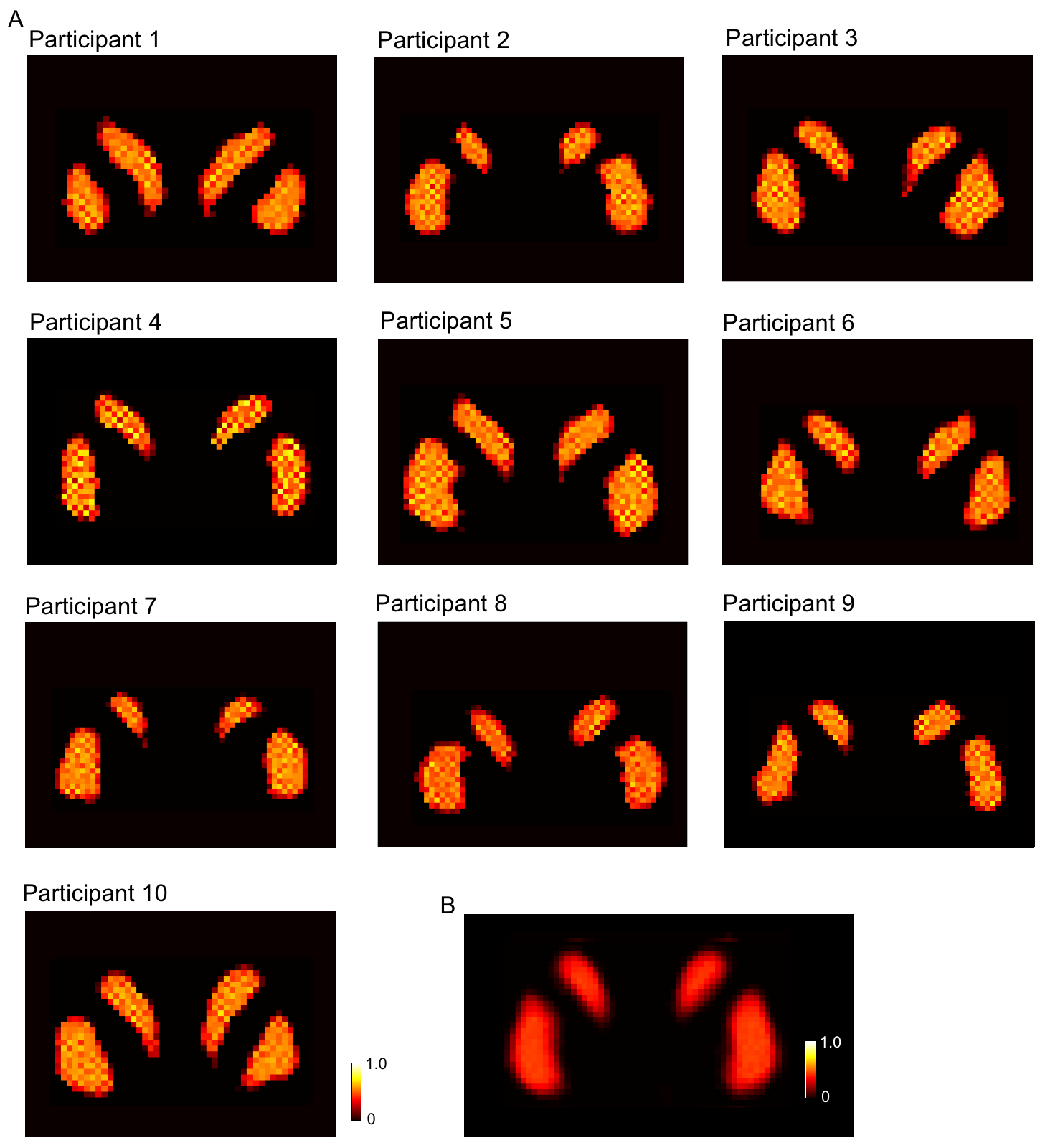


**Figure S2**  Striatal parcellation results. A) results of each individual. B) group results.


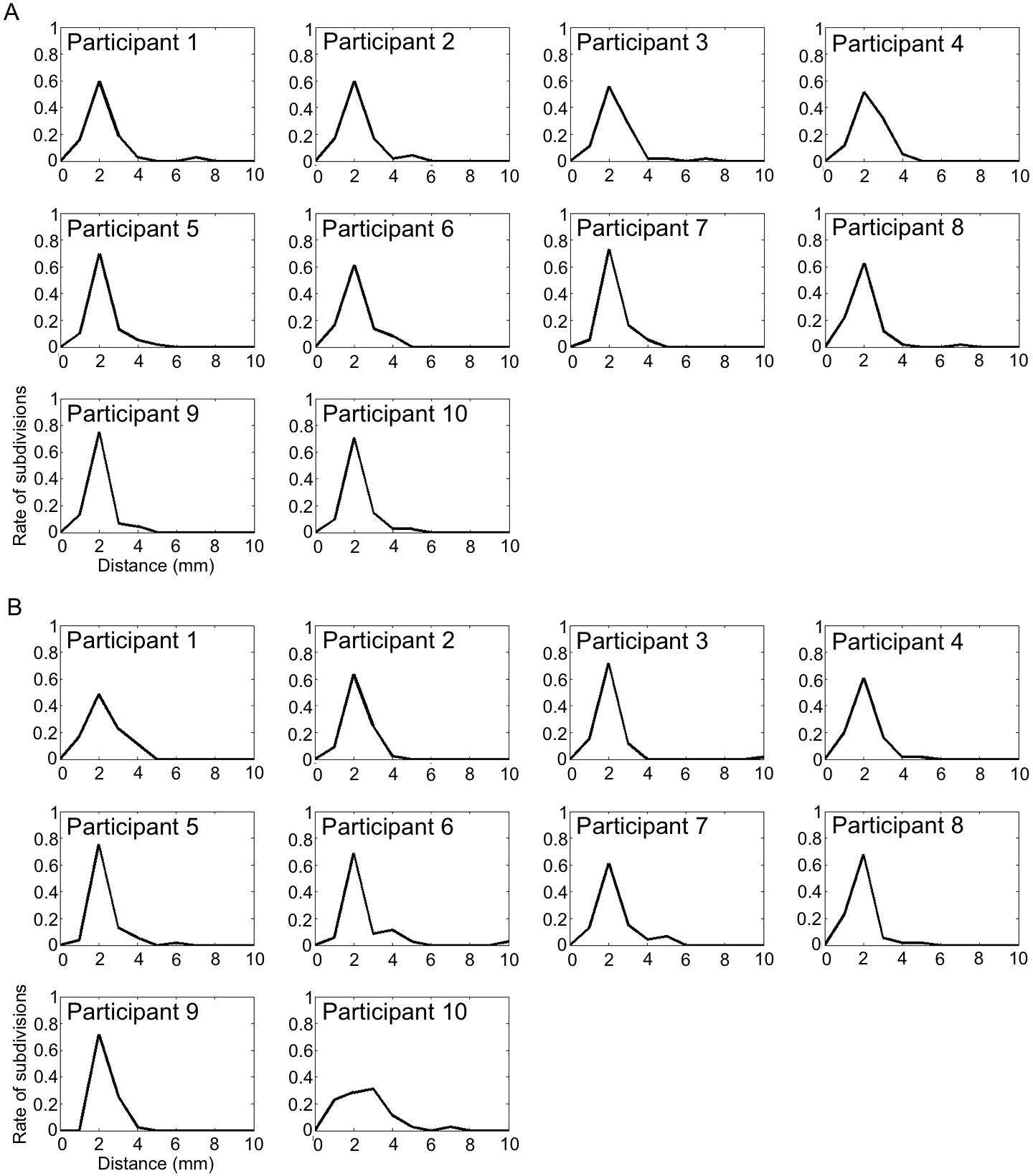


**Figure S3** Distribution of distance between adjacent striatal subdivisions. The ordinate indicates the probability normalized such that total value is 1. The abscissa indicates the distance with 1 mm bin width (e.g., 2 mm indicates 1.5 to 2.5 mm). (A) Left striatum. (B) Right striatum.

**
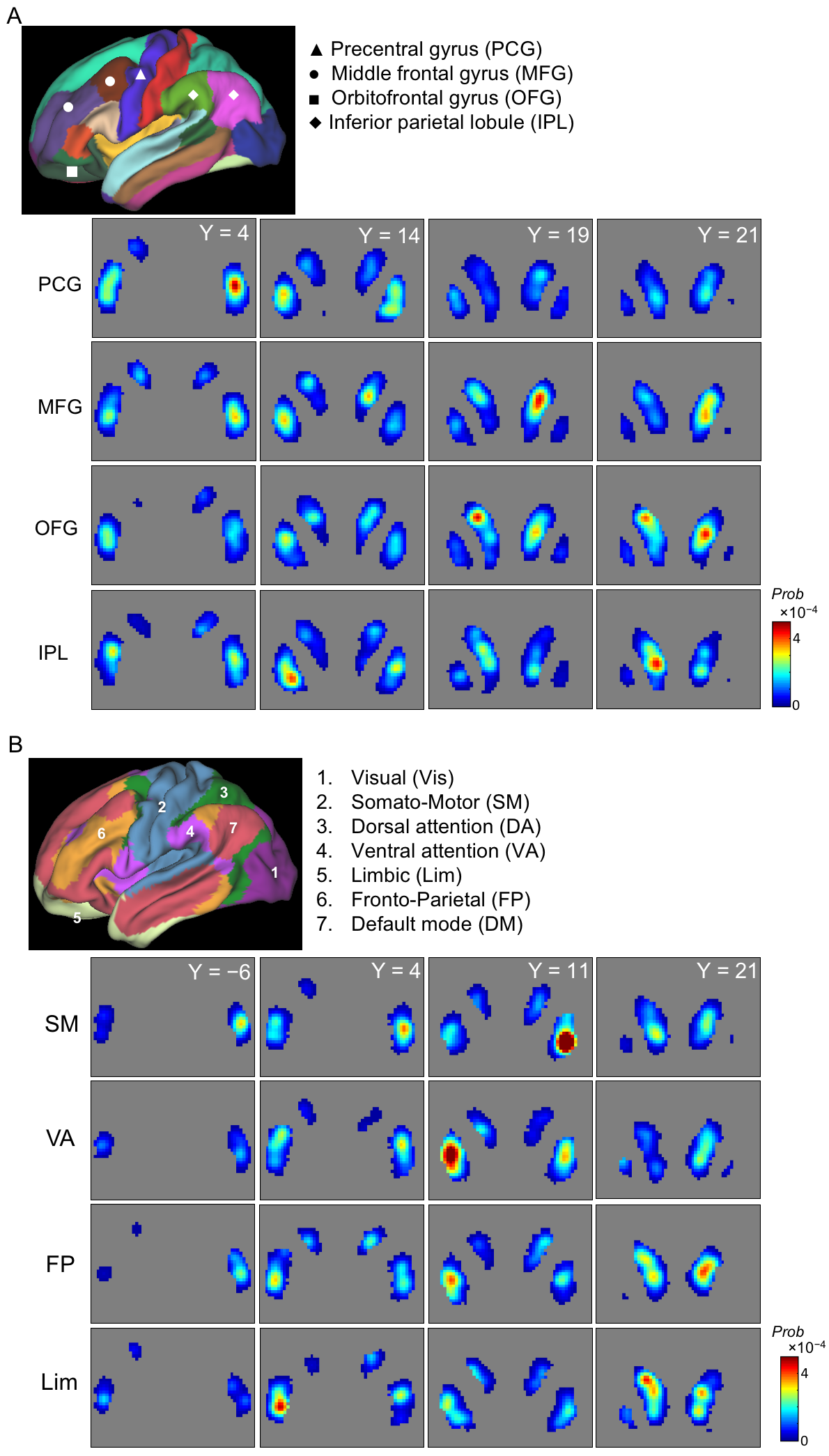
**

**Figure S4** Larger-scale striatal regions connected with cerebrocortical gyri and networks

(A) Group-level maps of probability of SSCs connected most strongly with the PCG, MFG, OFG or IPL. Color scale indicates probability of the map. PCG: precentral gyrus; MFG: middle frontal gyrus; OFG: orbitofrontal gyrus; IPL: inferior parietal lobule. (B) Group-level maps of probability of SSCs connected most strongly with the SM, VA, Lim or FP network. Vis: visual; SM: somato-motor; DA: dorsal attention; VA: ventral attention; Lim: limbic; FP: fronto-parietal; DM: default mode.


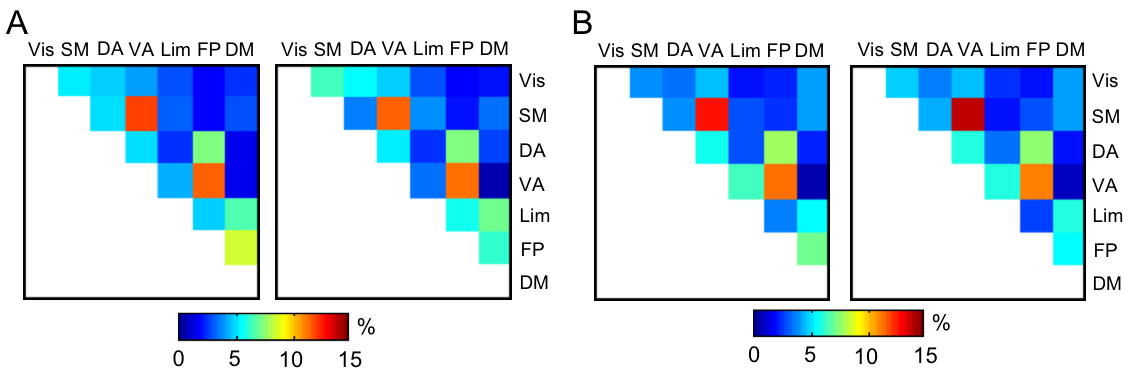


**Figure S5** Combinations of cerebrocortical networks connected with striatal subdivisions. (A) The distribution of network combinations when the second strongest connectivity was more than 50 % (left) and 70 % (right) of the strongest connectivity. (B) The distribution of network combinations when the size of the networks was reduced to approximately one half and when the second strongest connectivity was more than 50 % (left) and 70 % (right) of the strongest connectivity.

**
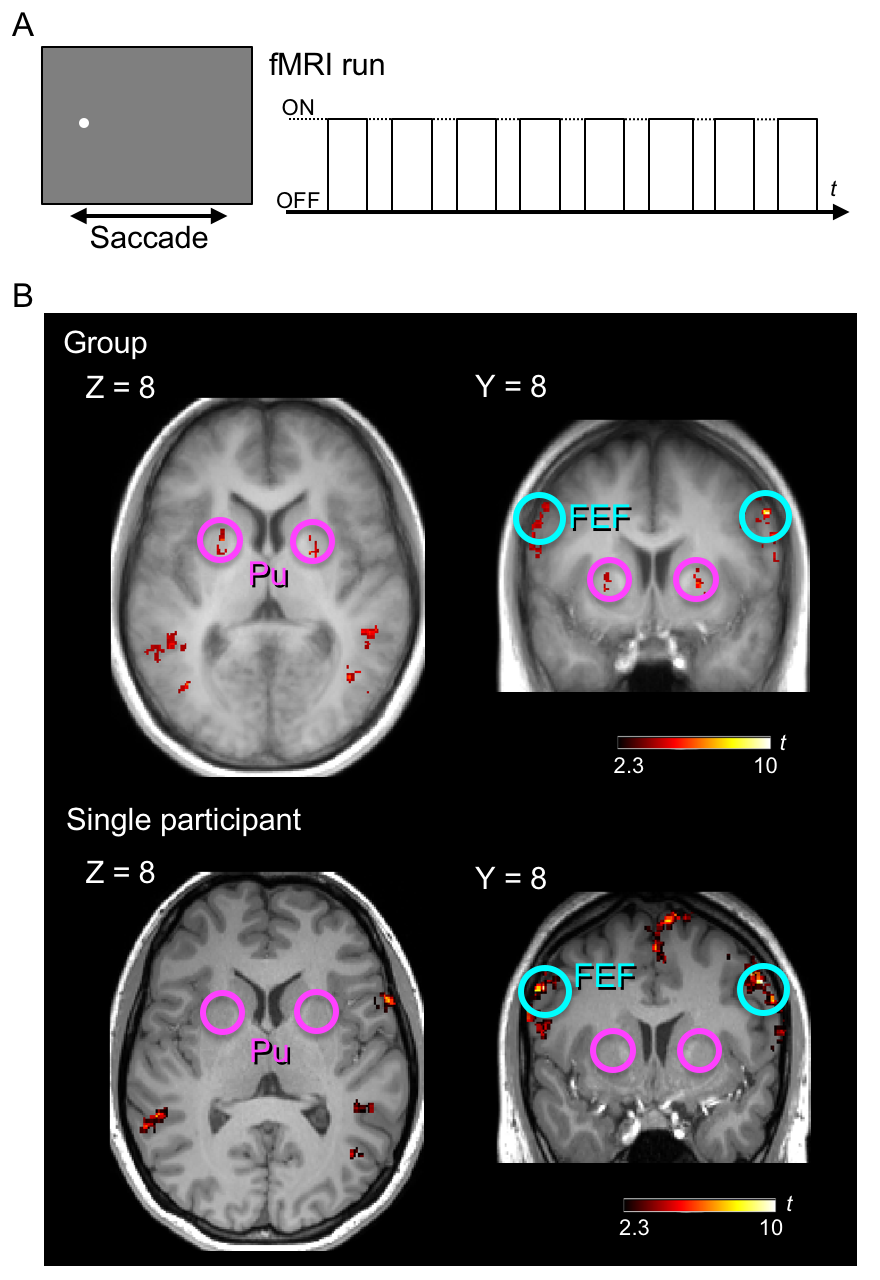
**

**Figure S6** Eye movement task to measure brain activity. (A) The blocked design of alternation of eye movement and fixation blocks. (B) Statistical brain activation maps in group-level and single-level analyses.

**Supplementary text 1**

Eight out of the ten participants also performed an eye movement task for 6 runs under the same scanning parameters used in the resting-state scans. The task was to make visually guided saccades, and the participants were instructed to follow a fixation point on the screen. The fixation point alternately moved leftward and rightward in 2 Hz during ON blocks, while the fixation point was presented in the center of the screen during OFF blocks. In the ON blocks, movement of the fixation point ranged from 3.5° to 17° from the center of the screen. Each run consisted of 8 ON blocks of 32 sec and 8 OFF blocks of 16 sec. During the scan, participants’ left eye was monitored to ensure that the participant performed the task.

Functional images of the activation data were preprocessed using SPM8 (www.fil.ion.ucl.ac.uk/spm/). The images were realigned, corrected for slice timing, and spatially smoothed with a 1.25-mm FWHM kernel. Saccade blocks were coded as a box car convolved with the canonical hemodynamic response function, and head movements were also coded as nuisance regressors. A single-level voxel-wise GLM analysis was performed to estimate signal magnitude and to evaluate statistical significance. After normalization to the standard template image using DARTEL, a group-level analysis was also carried out, using voxel-wise one-sample t-tests.
